# Emerging therapies for glioblastoma: current state and future directions

**DOI:** 10.1186/s13046-022-02349-7

**Published:** 2022-04-15

**Authors:** Liang Rong, Ni Li, Zhenzhen Zhang

**Affiliations:** 1 Institute of Human Virology, Key Laboratory of Tropical Diseases Control Ministry of Education, Zhongshan School of Medicine, Sun Yat-sen University, Guangzhou, China; 2 Key Laboratory of Brain, Cognition and Education Science, Ministry of Education, Institute for Brain Research and Rehabilitation, South China Normal University, Guangzhou, China

**Keywords:** Glioblastoma, Immunotherapy, Immune checkpoint blockade, CAR T, Oncolytic virotherapy, Vaccine, Focused ultrasound

## Abstract

Glioblastoma (GBM) is the most common high-grade primary malignant brain tumor with an extremely poor prognosis. Given the poor survival with currently approved treatments for GBM, new therapeutic strategies are urgently needed. Advances in decades of investment in basic science of glioblastoma are rapidly translated into innovative clinical trials, utilizing improved genetic and epigenetic profiling of glioblastoma as well as the brain microenvironment and immune system interactions. Following these encouraging findings, immunotherapy including immune checkpoint blockade, chimeric antigen receptor T (CAR T) cell therapy, oncolytic virotherapy, and vaccine therapy have offered new hope for improving GBM outcomes; ongoing studies are using combinatorial therapies with the aim of minimizing adverse side-effects and augmenting antitumor immune responses. In addition, techniques to overcome the blood-brain barrier (BBB) for targeted delivery are being tested in clinical trials in patients with recurrent GBM. Here, we set forth the rationales for these promising therapies in treating GBM, review the potential novel agents, the current status of preclinical and clinical trials, and discuss the challenges and future perspectives in glioblastoma immuno-oncology.

## Background

**Fig. 1:**
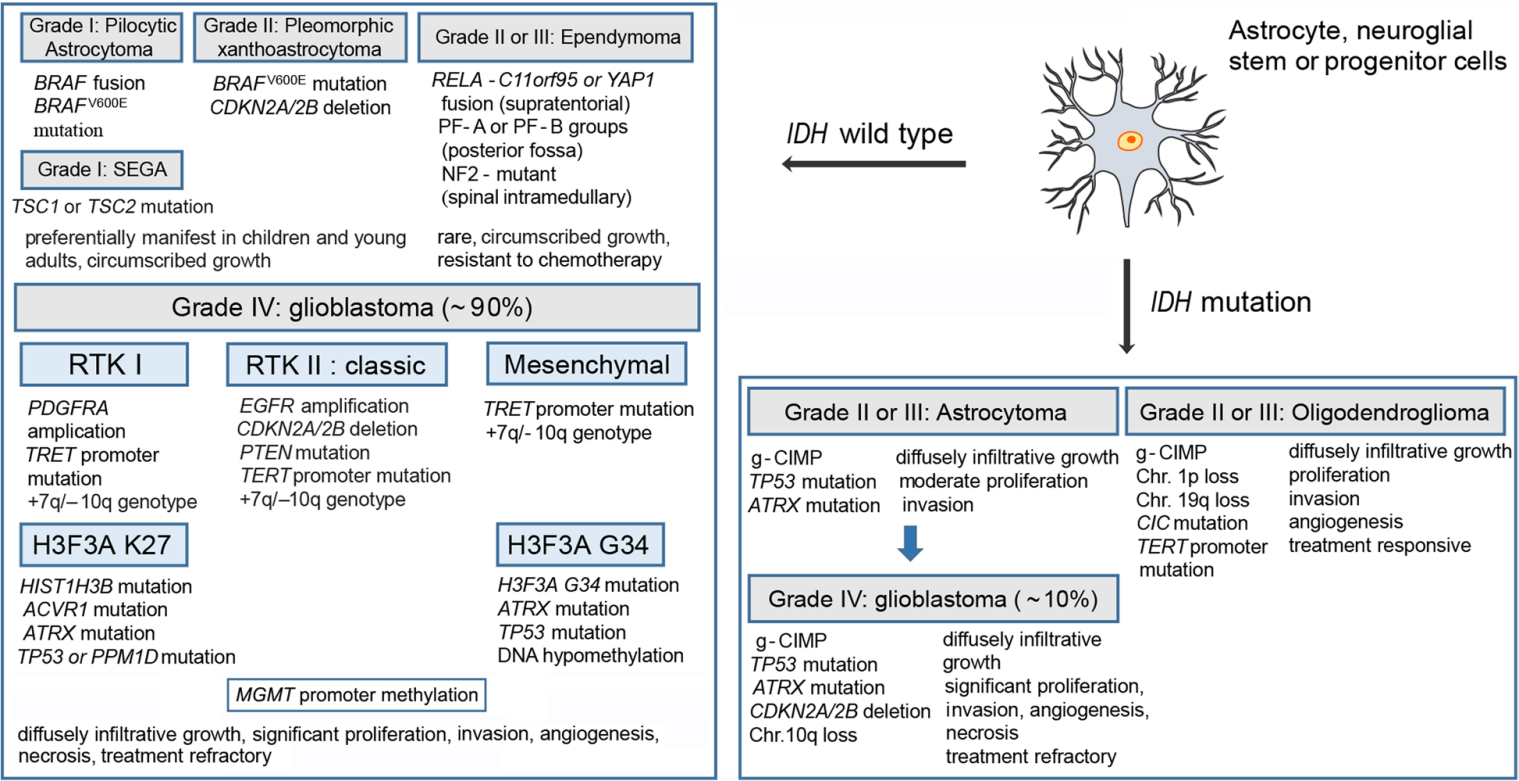
Genetic and epigenetic alterations in the genesis of gliomas. Shown are the relationships between the molecular lesions and pathobiology in the different types of gliomas. *IDH*, socitrate dehydrogenase; *RELA*, transcription factor p65; *CDKN*, cyclin-dependent kinase inhibitor; *YAP1*, YES-associated protein 1; PF, posterior fossa; *NF2*, neurofibromin 2; SEGA, subependymal giant cell astrocytoma; *TSC*, tuberous sclerosis; RTK, receptor tyrosine kinase; *PDGFRA*, platelet-derived growth factor receptor-α; *TERT*, telomerase reverse transcriptase; *PTEN*, phosphatase and tensin homologue; *EGFR*, epidermal growth factor receptor; *H3F3A*, histone H3.3; *HIST1H3B*, histone H3.1; *ACVR1*, activin A receptor 1; *ATRX*, α-thalassemia/mental retardation syndrome X-linked; *TP53*, tumour protein p53; *PPM1D*, protein phosphatase 1D; *MGMT*, O-6-methylguanine-DNA methyltransferase; g-CIMP, glioma CpG island methylator phenotype; Chr., chromosome; *CIC*, *Drosophila* homologue of capicua; Those *IDH*-mutant glioblastomas derived by progression from pre-existing lower grade astrocytomas (blue arrow) are tend to manifest in younger patients (≤50 years of age) compared with *IDH* wild-type tumors

Gliomas account for almost 30% of primary brain tumors and 80% of all malignant ones. Based on their histopathological features, gliomas are traditionally classified by the World Health Organization (WHO) as grade I and II (low-grade gliomas), grade III (anaplastic) and IV (glioblastoma) [1], which indicate different degrees of malignancy. In recent years, with the development of genomic, transcriptomic and epigenetic profiling, substantial advances have been achieved in new concepts of classifying and treating gliomas [2–6] (Fig. 1), which will complement the morphology-alone-based classification. The classification of molecular subtypes within the glioma facilitates molecular diagnosis in a timely manner to offer opportunities to select the proper treatment modality according to the demand of clinical practice [7]. Glioblastoma (GBM) is the most common and aggressive type of primary brain tumors, which comprises up to 50% of all gliomas. Despite progress made in the current standard of care including surgery, radiotherapy, and pharmacotherapy (typically chemotherapy with concomitant temozolomide (TMZ)), the outcome for patients remains almost universally lethal [2], with a median overall survival (OS) ranging from 14.6 to 20.5 months [8–12]. The prognosis is much worse in elderly patients, who have an average survival from diagnosis of less than 8.5 months [13]. Given the poor survival with currently approved treatments for GBM, new therapeutic strategies are urgently needed.

For most patients with GBM, there is no known causative factors for this disease. The only well-established exogenous environmental cause of glioma is exposure to high doses of ionizing radiation [14, 15]. Other risks including viral triggers (human cytomegalovirus) [16], obesity during adolescence [17], and family history of cancer [18] are continuing to be explored. Recent research has focused on identifying germline polymorphisms associated with risk of glioma, and reveals that genetic factors determine the degree of risk from these exposures [15]. Despite much efforts, little progress has been made in the survival outcomes of patients with GBM. The treatments fail mainly due to the unique molecular characteristics of GBM. Especially, the presence of a population of stem-like cells called glioma stem cells (GSCs) with ability of self-renewal and tumorigenicity, making it resistant to chemotherapy and radiotherapy [19, 20]. GBM cells have the propensity to infiltrate/invade into the adjacent normal brain tissues of tumor and along blood vessels, which prevents complete resection of the tumor and limits the effect of local radiotherapy [21]. Other features of GBM contributing to poor prognosis include: 1) the existence of the blood-brain barrier (BBB), 2) the relative immune privileged status of the central nervous system (CNS). Thus, precise strategies based on tumor-intrinsic dominant signaling pathways and tumor-specific antigenic profiles may ultimately improve outcomes for GBM patients. Fortunately, advances in decades of investment in molecular pathogenesis of glioblastoma are rapidly translated into innovative clinical trials, utilizing improved genomic, epigenetic, transcriptomic and proteomic characterization of glioblastoma as well as the brain microenvironment and immune system interactions [22]. With these encouraging findings, immunotherapy including immune checkpoint blockade, chimeric antigen receptor T (CAR-T) cell therapy, oncolytic virotherapy and vaccine therapy have been actively tested in clinical trials for GBM [23]. Studies are ongoing to use combinatorial therapy with the aim of reducing adverse effects and enhancing antitumor responses [24–26]. Moreover, emerging insights into BBB features have yielded novel strategies to improve drug penetration into the tumor and infiltrative regions [27]. On the basis of preclinical work [28–33], focused ultrasound therapy have been tested in clinical trials and achieved improved treatment outcomes in patients with recurrent GBM [34, 35], opening avenues for the development of innovative combinatorial strategies for targeting GBM. Herein, we set forth the rationales for these promising therapies in treating GBM, review the potential therapeutic targets, the current status of pre-clinical and clinical trials, and discuss the challenges and future directions of emerging therapies.

### The CNS is an immunologically distinct site

Due to the presence of BBB, lack of dedicated lymphatic channels, low basal expression level of Major Histocompatibility Complex (MHC) class II molecules, paucity of antigen presenting cells (APC) and the constitutive expression of immunosuppressive cytokines such as TGF-ß, the CNS has long been considered as an immune-privileged site with restricted access that profoundly affects the capacity of T cells to exert their functions [36]. Consistent with this, high level of TGF-β was observed in intracranial gliomas in experimental models, leading to accumulation of both Tregs and immature dendritic cell (DC). This milieu prevented T-cell priming and re-stimulation, and ultimately impaired anti-tumor immune response [37]. However, more recent findings have improved our understanding of immunological mechanisms in the CNS. In 2015, Louveau et al. defined a classical lymphatic system in the CNS, which are able to carry both fluid and immune cells from the cerebrospinal fluid [38]. Thus, most antigen-presenting cells exiting the brain can travel to the deep cervical lymph nodes to prime T and B lymphocytes, indicating that immunogens present in the brain are capable of generating adequate immune responses [38]. Consistent with these findings, clinical data showed that downregulation of human leukocyte antigen (HLA) class I expression corresponds with poor prognosis in GBM [39] and low CD4^+^ T cell counts correlate with adverse outcomes in patients receiving conventional therapy for high-grade gliomas [40]. Regarding T lymphocytes, CD8^+^ T cells infiltrating in newly diagnosed glioblastoma was reported to prolong the survival of patients [41]. Taken together, these observations implicate that a T-cell response to GBM could potentially modulate outcome [36]. On the basis of evidence from preclinical and clinical studies, the CNS should more accurately be viewed as a unique immune environment. Immune reactions in the CNS are common, but take on a distinctive character, which is probably dictated by the natural microenvironment [42]. Normally, the CNS is immunologically quiescent in the healthy brain. In adults, microglia account for approximately 10% of CNS cells and maintain a quiescent phenotype in the normal CNS, expressing low levels of MHC molecules and costimulatory molecules [43]. Upon inflammatory conditions, peripheral leukocytes access the CNS and orchestrate immune responses, activated microglia upregulate MHC II molecules as well as costimulatory molecules and present antigens to activated lymphocytes [44], providing the fundamental basis for immunotherapy directed against brain tumors. Collectively, these findings support the notion that, while the brain is an immunologically specialized site, the immune microenvironment offers opportunities to develop immunotherapy for the treatment of GBM [45].

### Current standard of care and immunotherapy

GBM is currently incurable because of its high recurrence after standard multimodality treatment, including surgery to remove the main tumor followed by concomitant radiation and adjuvant TMZ chemotherapy to target residual tumor cells. Because of the presence of GSCs, it requires complete destruction of the tumor, even a miniscule amount of residual tumor can lead to fatal recurrence [46]. Recently, intraoperative imaging techniques to maximize extent of resection have contributed considerably in defining the margins of glioblastoma [47, 48]. However, radical extirpation of the tumor is not possible due to infiltration of the tumor into the surrounding brain, the role of image-guided surgery in maximizing extent of resection remains uncertain [46]. Currently, TMZ replaced nitrosoureas as the standard for patients with newly diagnosed GBM. To a certain extent, the success of this strategy depends on the methylation status of O-6 methylguanine-DNA methyl-guanine-methyltransferase (*MGMT*) [13]. In agreement to this, a Phase III trial demonstrated that GBM patients with *MGMT* promoter methylation achieved higher survival rates than patients with unmethylated *MGMT* promoter [49]. Subsequently, a randomized Phase III trial (NCT00006353) in elderly GBM patients confirmed that patients with *MGMT* promoter methylation benefitted more from adjuvant TMZ with radiotherapy than radiotherapy alone [49], suggesting that the benefit seen in patients with *MGMT* promoter methylation may possibly correlated to addition of TMZ. Radiotherapy remains the primary treatment modality in unresectable GBM. Radiotherapy is usually combined with chemotherapy following surgery in different sequential combinations. According to a systematic review of randomized clinical trials, radiotherapy plus TMZ provides better survival outcomes than radiotherapy alone in treating GBM [50]. Recently, A multi-institutional GBM-molRPA cohort reported that conventionally fractionated standard radiotherapy significantly prolonged OS than short-course radiotherapy in selected elderly GBM patients treated with TMZ-based chemoradiation [51]. Given that TMZ can presents unwanted systemic toxicity, combination strategies with the aim of reducing adverse effects and augmenting anti-tumor responses are urgently needed. Recently, in an open-label, randomized, phase III trial (NCT01149109), combined lomustine-TMZ chemotherapy prolonged overall OS survival compared with standard adjuvant therapy in patients with newly diagnosed glioblastoma with methylated *MGMT* promoter [52], providing new evidence that dual agent treatment may be superior to TMZ alone for GBM [53]. Despite multimodal therapies, the prognosis of GBM is still disappointing. To a certain degree, distinction of molecular subtypes within the glioma (Fig. 1) offer possibilities to select the proper treatment modality according to the demand of clinical practice. However, to date, the classification scheme is of limited relevance for GBM treatment due to intratumoral heterogeneity.

Immunotherapy, which harnesses the body’s immune system to against cancer, has led to important clinical advances over the past few years [54–56]. On the basis of therapeutic gains made in immune checkpoint blockade and CAR-modified T cells, Science awarded cancer immunotherapy its ‘Breakthrough of the Year’ in 2013 [56]. Subsequently, The Nobel Prize in Physiology or Medicine 2018 awarded discovery of cancer therapy by inhibition of negative immune regulation. These excellent findings laid the foundation for the clinical development of immunotherapy, which have dramatically improved outcomes for many people with cancer. In recent years, lots of immunotherapy drugs, from monoclonal antibody against cytotoxic-T-lymphocyte-associated protein 4 (CTLA-4), programmed cell death protein 1 (PD-1) and PD-1 ligand 1 (PD-L1), to CAR T cell therapy, are approved by U.S. Food and Drug Administration (FDA) for cancer treatment [54–57]. Although no FDA-approved immunotherapies for GBM exists currently, there are several ongoing clinical trials testing in GBM patients, spurred on by advances in immuno-oncology for other tumor types [58]. Recently, treatment with immune checkpoint inhibitors demonstrated improved OS in some melanoma patients with brain metastases, suggestive of the immunotherapy as a potential treatment option for CNS tumors [59, 60]. Despite this, a persistent challenge remain for immunotherapy in treating GBM due to the existence of redundant mechanisms of tumor-mediated immune suppression [61, 62]. Besides, molecular heterogeneity in GBM is credited as a major mechanism of therapeutic resistance and therefore an important clinical challenge to develop effective immunotherapeutic directed against GBM [63]. In addition, adverse events (AEs) with immune-mediated mechanisms are common in patients with advanced solid organ malignancies receiving immunotherapy [64]. Based on these observations, advancements in immunotherapy for GBM is an exciting direction for the future development of treatments for GBM, but their clinical benefits remain to be seen [63].

### Immune checkpoint blockade

Immune checkpoints exist to dampen or terminate immune activity to guard against autoimmunity and maintain self-tolerance, acting as so-called ‘brakes’ on the immune system. However, tumors can co-opt immune checkpoint pathways to evade immune surveillance. Drugs targeting immune checkpoints, such as CTLA-4, PD-1, and PD-L1 can enhance anti-tumor immune responses and allow T cells to more effectively eradicate cancer cells. Given the success with many solid tumors, the potential of immune checkpoint blockade therapy, has been actively pursued for GBM. Nonetheless, GBM harbour a relatively low number of somatic mutations and lack T-cell infiltration compared with other tumor types [65], which may limit the availability of immune checkpoint blockade. In this regard, GBM is thought as a type of “cold tumor”. Still, immune checkpoint inhibitors have garnered considerable interest for the treatment of GBM, considering the unique immunologically properties of CNS.

CTLA-4 and PD-1 are negative regulators of T-cell activity that limits immune responses against cancer [56]. PD-1 binds to its ligands PD-L1, which is expressed in GBM tumors [66, 67], and elevated expression levels was shown to correlate with poorer prognoses in some studies [67]. Ipilimumab is a human anti-CTLA-4 monoclonal antibody (mAb) that blocks CTLA-4 and its ligands (CD80/CD86) with demonstrated efficacy in metastatic melanoma [68]. Preclinical research has suggested that the combination of CTLA-4 and IL-12 blockade elicits T cell-mediated glioma rejection in a syngeneic murine model of GBM [69]. A durable survival benefit was achieved utilizing combinatorial blockade against CTLA-4, PD-L1 and indoleamine 2,3 dioxygenase 1 (IDO) in glioma-bearing mice models [70]. The nivolumab is a fully human immunoglobulin G subclass 4 monoclonal antibody inhibitor of PD-1 approved globally for the treatment of diverse cancers [71]. Growing studies have demonstrated that the PD-1/PD-L1 axis is immunologically relevant and a therapeutic window exists [72–74]. Taken together, these data provide preclinical evidence that combinatorial targeting immunosuppression may serves as a promising strategy for future clinical trials in patients with GBM. Since immune checkpoint blockade have revolutionized cancer treatment for several solid tumors, there exists the possibility that it can also transform the treatment of GBM. Based on these findings, an early phase I study evaluated the safety/tolerability and efficacy of nivolumab alone or in combination with ipilimumab for patients with recurrent glioblastoma [75]. In this trial, 40 patients were enrolled from 9 sites in the United States, and exploratory efficacy results indicated that ~ 20% of patients achieved stable disease ≥12 weeks, and 5 (12.5%) survived > 25 months. Additionally, nivolumab monotherapy was better tolerated than nivolumab in combination with ipilimumab and was selected for the phase III cohort (cohort 2) of (CheckMate 143, NCT02017717). It should be note that high rates of serious adverse events were observed in nivolumab with ipilimumab, thus this combination strategy is not being pursued further in the phase III stage of this trial. In this phase III trial, the efficacy and safety of nivolumab is being compared with that of bevacizumab (a monoclonal antibody to vascular endothelial growth factor) in patients with recurrent glioblastoma, the preliminary data reported at the 2017 World Federation of Neuro-Oncology Societies meeting revealed that at interim analysis of 369 patients, nivolumab monotherapy did not demonstrate a median OS benefit over bevacizumab (9.8 months with nivolumab versus 10.0 months bevacizumab) [45]. Although the study did not met the primary end point of OS, no safety concerns were reported [76]. The results also revealed that patients with methylated *MGMT* promoter and no baseline corticosteroid dependence may potentially derive benefit from treatment with immune checkpoint blockade [76]. In a large ongoing randomized phase II trial (CheckMate 548, NCT02667587), researchers are investigating nivolumab as an alternative to TMZ (both in combination with radiotherapy) in newly diagnosed GBM patients with methylated *MGMT* status. A similar ongoing phase III trial for patients with unmethylated *MGMT* status will also be assigned to receive nivolumab + standard radiotherapy vs. TMZ + standard radiotherapy (CheckMate 498, NCT02617589) [77]. Although the results from these two trials are unpublished at this time, the preliminary data stated by Bristol-Myers Squibb (BMS) at 2019 revealed that CheckMate 548 did not meet one of its primary endpoints and CheckMate 498 did not meet its primary endpoint of OS on final analysis [78]. Furthermore, a single-arm phase II clinical trial in which neoadjuvant nivolumab was tested in 30 patients with recurrent resectable glioblastoma observed favorable changes in the tumor immune microenvironment (NCT02550249) [79]. Although no obvious clinical benefit was substantiated following salvage surgery, two of the three patients treated with nivolumab before and after primary surgery remain alive 33 and 28 months later [79]. Moreover, a small randomized phase II clinical trial in this same issue [80], utilizing neoadjuvant pembrolizumab (a humanized monoclonal antibody that binds the PD-1 receptor) in patients with recurrent resectable glioblastoma described similar intratumoral effects in the immune tissue microenvironment as evidenced by [79]. The neoadjuvant administration of PD-1 blockade enhances the local and systemic anti-tumor immune response and may provide a therapeutic window to study the immunobiology of GBM [80]. Admittedly, these two studies was a small study in which the limited sample size prevents definitive conclusions about the clinical outcome of treatment. To date, clinical trials have revealed that immune checkpoint inhibitors have limit efficacy in GBM, where < 10% of patients show long-term responses. The main reason might be that multiple genomic features are involved in the occurrence and development of GBM, which may determine the response pattern of patients with GBM to checkpoint blockade immunotherapy. To understand the molecular determinants of immunotherapeutic response in GBM, a recent study enrolled 66 patients to investigate the immune and genomic correlates of response to anti-PD-1 immunotherapy in GBM. Genomic and transcriptomic analysis revealed that *PTEN* mutations are associated with immunosuppressive expression signatures and resistance to immune-checkpoint inhibition, whereas tumors from responders were observed to harbour MAPK pathway alterations (*PTPN11*, *BRAF*) [81]. Of note, a survival difference was seen between responders and non-responders, with a median survival of 14.3 months of responders compared to the 10.1 months of non-responsive patients. Whereas thousands of unselected patients received immune checkpoint inhibitors without evidence of significant response to date, this study showed that a sub-group of patients might benefit from this therapy, suggesting a possibility of personalized, patient-specific GBM treatment.

Beyond that, case reports suggested the effective of anti-PD-1 monotherapy for patients with GBM. Two pediatric patients with recurrent multifocal GBM refractory to current standard therapies exhibited impressive and durable responses to nivolumab [82]. In addition, an adult patient with germline *POLE* deficiency who developed a hypermutated glioblastoma showed a clinical response to pembrolizumab [83]. Notably, the patients with high tumor mutational loads are thought to respond well to immune checkpoint inhibitors in these two reports. Furthermore, in an adult patient with recurrent GBM, treatment with nivolumab resulted in long-term disease control without needing further steroid medication [84]. While these findings are encouraging, phase III clinical have not demonstrated a clear benefit for single checkpoint inhibitor and no FDA-approved immunotherapy for GBM exists [58]. Clinical trials outside of GBM have uncovered that a number of biomarkers predict clinical responses to PD-1 axis blockade in cancer therapy [85]. Well characterized biomarkers including tumor mutational burden [86] and PD-L1 expression [87] have been identified in diverse cancer types. Given that the extent of PD-L1 expression in GBM remains the subject of debate [45], and GBM is typically have a relatively low mutational burden in most cases, a detailed evaluation of validated biomarkers for patient selection and disease surveillance may be particularly important for GBM immunotherapy.

### On the horizon: targeting “next-generation” checkpoints

Although CTLA-4 and PD-1 blockade are the focus of the basic research and clinical attention, continued exploration of additional checkpoints may lead to development of combination treatment strategies that can improve responses and expand immune checkpoint blockade to a greater number of GBM patients [58, 77].

### CD47

Unlike the adaptive immune checkpoint PD-L1 who sends to the adaptive immune system a “don’t find me” signal, cluster of differentiation 47 (CD47) sends a “don’t eat me” signal to the innate immune system that blocks macrophages from attacking the tumor [88, 89]. The binding of CD47 to its cognate receptor signal-regulatory protein alpha (SIRPα) on phagocytic cells leads to inhibition in the macrophage-mediated tumor cell phagocytosis. Since the recent identification of CD47/SIRPα axis as a therapeutic target for human solid tumors [90–92], there have been several preclinical studies examining the safety and efficacy of targeting CD47 as an immune checkpoint molecule for GBM therapy [93, 94]. CD47 blockade by using anti-CD47 antibody was reported to stimulate phagocytosis of glioblastoma by macrophages and hence reduce tumor burden in vivo [95]. In vitro and in vivo studies demonstrated that CD47 blockade by a humanized anti-CD47 antibody (Hu5F9-G4) enhanced macrophage-mediated phagocytosis, improved survival, and reduced tumor burden in human GBM engrafted mice model [92]. In vivo study further showed that CD47 blockade could effectively reeducate microglia in the GBM tumor microenvironment to unleash the therapeutic potential of tumor cell phagocytosis [96]. Additionally, anti-CD47 immunotherapy using Hu5F9-G4 could also be combined with irradiation or TMZ chemotherapy to enhance the therapeutic efficacy of GBM treatment in vitro and in vivo [97]. Recently, a pre-clinical toxicokinetic study in non-human primates reported no adverse effects associated with Hu5F9-G4. As a matter of fact, CD47 therapeutics including Hu5F9-G4 are moving forward rapidly in the clinic, patients with acute myeloid leukemia (AML) and solid tumors are being recruited for phase I clinical trials (NCT02678338 and NCT02216409) [98, 99]. A phase 1b study demonstrated that the combination of Hu5F9-G4 and rituximab produced durable responses in patients with aggressive and indolent lymphoma. No clinically significant safety events were observed in this initial study (NCT02953509) [100], and further investigation is ongoing in a phase II trial (NCT02953509). However, no clinical trials have been conducted in GBM to date, the development of CD47 blockade as a therapeutic target either as monotherapy or in combination with other treatments for GBM needs to be studied further.

### CD73

Despite the achievements of immune checkpoint blockade therapy in advanced cancer, a considerable proportion of patients remain unresponsive to these treatments, suggestive of multiple non-redundant immunosuppressive mechanisms coexist within the tumor microenvironment. One such mechanism is the conversion of inflammatory extracellular adenosine triphosphate (ATP) into immunosuppressive extracellular adenosine (eADO) [101, 102]. The canonical pathway is started from the hydrolysis of ATP to AMP by CD39 (also known as ectonucleoside triphosphate diphosphohydrolase 1); non-canonical pathway metabolizes NAD^+^ to ADP-ribose (ADPR) through CD38, which is then processed to AMP by ectonucleotide pyrophosphatase/phosphodiesterase family member 1 (CD203a/PC-1) [103]. Both pathways converge to CD73 (also known as 5′-nucleotidase), that fully degrades AMP to the final product ADO. Targeting CD73 on host and tumor cells was shown to alleviate adenosine-mediated immunosuppression and to inhibit tumor progression in human solid tumor models [104, 105]. Preclinical research revealed that targeted blockade of CD73 could enhance the therapeutic activity of anti-PD-1 and anti-CTLA-4 monoclonal antibody [106]. Across various cancer types, high expression of CD73 has consistently correlated with poor prognosis in patients, thus justifying the rationale to target CD73 in the clinic [102]. Currently, novel agents targeting the adenosinergic pathway are now reaching clinical trials in patients with advanced cancer either as single agents or in combination with conventional and immunotherapies [102].

MEDI9447 is a human monoclonal antibody that selectively binds to and inhibits the ectonucleotidase activity of CD73, and the results showed the ability of MEDI9447 in preclinical tumor models [107]. A phase I trial to test the safety, tolerability, and clinical activity of oleclumab (MEDI9447) alone and in combination with durvalumab (MEDI4736, anti-PD-L1) is currently underway (NCT02503774). Other anti-CD73 monoclonal antibodies, including BMS-986179 (NCT02754141), CPI-006 (NCT03454451) and NZV930 (NCT03549000) have been also actively tested in clinical trials [101]. Aside from mAbs, novel small-molecule inhibitors of CD73 are being developed and tested as monotherapy or in combination with other immunotherapies in preclinical and clinical trials, such as A001421 [94], AB680 (NCT04104672), CB-708, and LY3475070 (NCT04148937) [101]. A001421 have demonstrated profound effects in experimental tumor models when dosed in combination with PD-1 blockade [102]. Overall, the therapeutic efficacy of these CD73 blockade in patients with cancer is eagerly awaited. With regards to GBM, Preclinical studies showed that CD73 blockade decreases in vitro and in vivo glioblastoma growth and potentiates TMZ induced glioma cytotoxicity [108]. Further study revealed that blockade of CD73 delays glioblastoma growth by modulating the immune environment [109]. Of note, immune profiling data from multiple different human tumors and an anti-PD-1 clinical trial in patients with GBM identified CD73 as a specific immunotherapeutic target to improve outcomes for immune checkpoint therapy in glioblastoma multiforme [110]. The absence of CD73 significantly improved survival in a murine model of glioblastoma multiforme treated with anti-CTLA-4 and anti-PD-1 [110], suggestive of CD73 as a combinatorial target in glioblastoma. Although this reverse translational study provide some positive findings of combination immune-checkpoint blockade, it remains to be seen whether the preclinical response observed with combination immune-checkpoint blockade will translate to clinical trials in patients with GBM [111].

### CAR T therapy

**Fig. 2:**
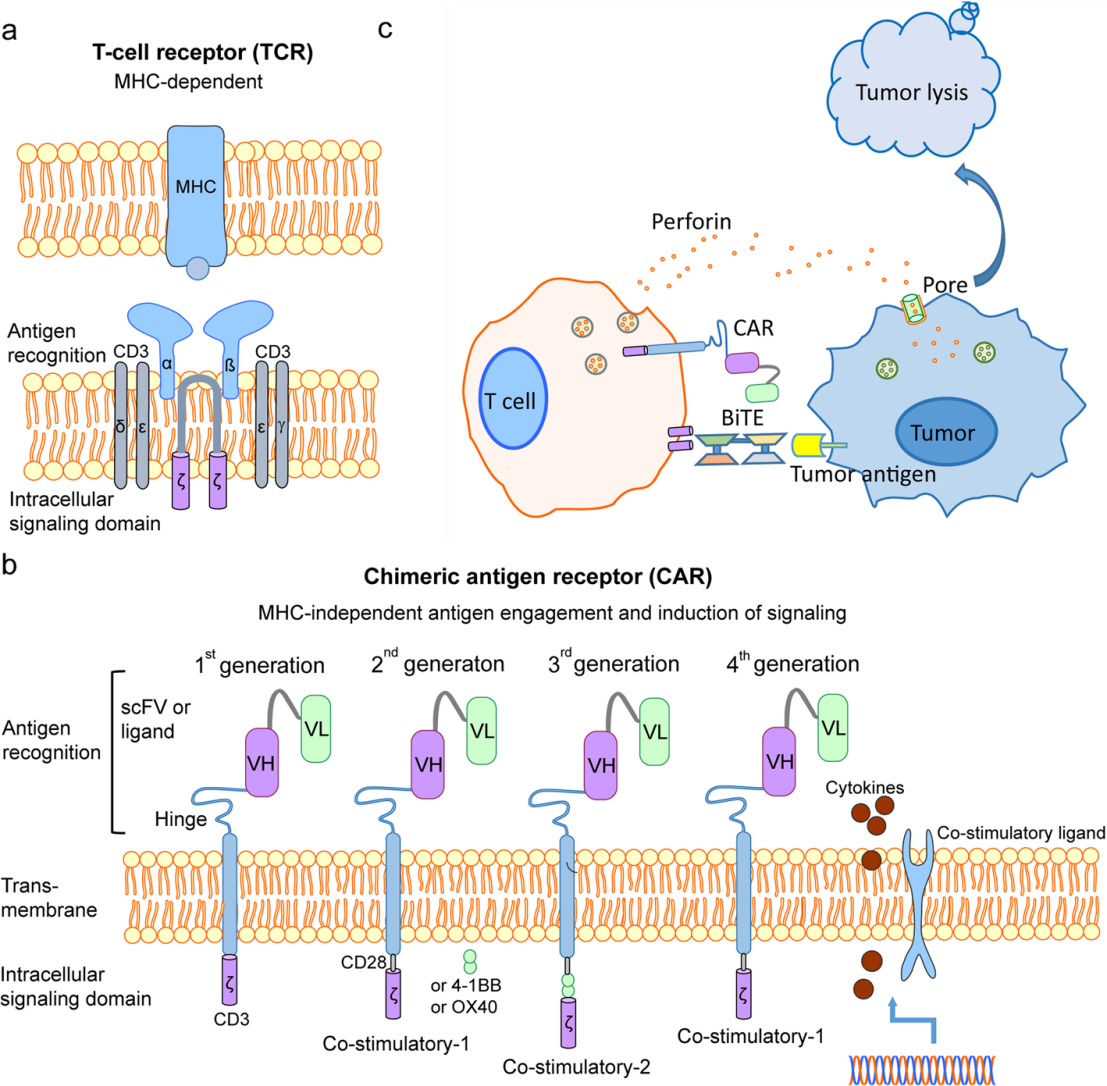
General structure of CAR and CAR T-cell therapy. **a** Basic structure of T-cell receptor (TCR). The TCR comprise variable TCR-α and -β chains coupled to three dimeric signaling transduction modules CD3 δ/ε, CD3 γ/ε and CD3 ζ/ζ. T cell activation usually requires MHC matching. **b** Structure of 1st- 4th generation CARs. Chimeric antigen receptor (CAR) are fusion proteins consisting of an extracellular domain with a tumor-binding moiety, typically a single-chain variable fragment (scFv), followed by a hinge of varying length and flexibility, a transmembrane (TM) region, and one or more intracellular signaling domains associated with the T-cell signaling. First-generation CARs contain the stimulatory domain of CD3ζ, whereas second-generation CARs possess a co-stimulatory domain (typically CD28 or 4-1BB) fused to CD3ζ to ensure full activation. Third-generation CARs consist of two co-stimulatory domains linked to CD3ζ to maximize signaling activation. The first co-stimulatory domain is either a CD28 or a 4-1BB domain, with the second co-stimulatory domain consisting of either a CD28, a 4-1BB or a OX40 domain. The fourth-generation CARs, combine the second-generation CAR with the addition of various genes, including cytokines and co-stimulatory ligands, to enhance the tumoricidal effect of the CAR T cells. **c** Mechanisms of CAR-T therapy. CAR-T cells can produce an artificial T cell receptor that has high affinity to a tumor-specific surface antigen. BiTEs can redirect T cells to tumor cell surface antigens and activate T cells. Activated T cells release perforin and other granzymes through immunological synapses. These cytolytic proteins can form pores on tumor cell surface, and thus are endocytosed by tumor cells and then form endosomes and lyse tumor cells ultimately form endosomes in tumor cells and lyse tumor cells ultimately

Genetic engineering of T cells to express CARs directed against specific antigens of tumor cells has emerged as a promising new treatment for cancer therapy [112] (Fig. 2). CAR are chimeric constructs containing an extracellular domain with tumor-binding moiety, typically a single-chain variable fragment (scFv), followed by a hinge of varying length and flexibility, a transmembrane (TM) region, and one or more intracellular signaling domains associated with the T-cell signaling. First-generation CARs contain the stimulatory domain of CD3ζ, whereas second-generation CARs possess a co-stimulatory domain (typically CD28 or 4-1BB) fused to CD3ζ to ensure full activation. Third-generation CARs consist of two co-stimulatory domains linked to CD3ζ to maximize signaling activation. The first co-stimulatory domain is either a CD28 or a 4-1BB domain, with the second co-stimulatory domain consisting of either a CD28, a 4-1BB or an OX40 domain. The fourth-generation CARs, combine the second-generation CAR with the addition of various genes, including cytokines and co-stimulatory ligands, to enhance the tumoricidal effect of the CAR T cells [113–115] (Fig. 2). Once the modified T cells are administered into the patient, where they can initiate cytotoxic attack on the antigen-bearing tumor cell. Since CAR recognition is either independent on MHC or effective presentation of target epitopes, CAR T therapy has the advantage of bypassing the need for MHC presentation of antigen and development of adaptive immune response [115].

The clinical potential of CAR T-cell therapy has been most convincingly shown in the field of hematological malignancies [116–118]. Given their extraordinary efficacy in hematological malignancies, efforts have been made to apply CAR T-cell therapies for the treatment of solid tumors including GBM [113, 114, 119]. Recently, several clinical CAR T cell therapies have already been tested for GBM using epidermal growth factor receptor variant III (EGFRvIII), interleukin (IL)13Rα2 (IL-13Ra2), and ephrin-A2 (Her2) as targets, with mixed but informative results [120, 121]. Previous clinical study provide promising first-in-human clinical evidence for feasibility of intracranial administration of IL13Rα2-specific CAR T cells for the treatment of GBM, establishing the foundation for further development of this IL13Rα2-specific CAR T cell therapy [122]. Building on the initial results, Brown et al. [123] report a case study in which a patient with recurrent multifocal glioblastoma received CAR T cells targeting IL-13Rα2. After CAR T-cell treatment, regression of all intracranial and spinal tumors was observed, along with corresponding elevated levels of cytokines and immune cells in the cerebrospinal fluid [123]. The clinical response lasted for 7.5 months after the initiation of CAR T-cell therapy. While the exact cause of relapse remains to be elucidated, instances of tumor recurrence with loss and/or reduced expression of IL13Rα2 has been observed [122, 123]. However, this study indicates that in addition to directly targeting tumor cells via IL13Rα2, the CAR T- cells induce an endogenous immune response as increased levels of non-CAR T immune cells and cytokines was observed after each infusion and the treatment was successful in initial tumors despite IL13Rα2 escape. In addition, a phase I dose-escalation study of infusing HER2-CAR-modified autologous virus-specific T cells (VSTs) (HER2-CAR VSTs) in patients with progressive glioblastoma has been conducted [124]. The data showed that infusion of autologous HER2-CAR VSTs is safe and can be associated with clinical benefit for patients with progressive GBM [124].

In 2017, the results of a first-in-human clinical trial (NCT02209376) of CART-EGFRvIII in 10 patients with recurrent EGFRvIII-positive GBM were published [125]. The results demonstrated that manufacturing of CART-EGFRvIII cells from patients with recurrent GBM was safe and feasible. Although no survival benefit was observed from this small study, the authors found that CART-EGFRvIII cells infused intravenously did traffic to the brain tumor and exert antigen-directed activity [125]. In addition, lower EGFRvIII expressions and the inhibitory tumor microenvironment were also observed post-therapy [125]. Such antigen escape mechanisms may limit the durability of responses to CAR T therapy. Recently, bispecific T cell engagers (BiTEs) have been proposed as a solution against antigen escape (Fig. 2) [126]. By engineering EGFR-directed BiTEs, which tether T cells to tumor cells, into the EGFRvIII-CAR T cells, producing a dual-targeted platform to prevent antigen escape. EGFR-targeted BiTEs produced by CAR T cells demonstrated minimal toxicities and antitumor activity against heterogeneous tumors, highlighting a promising avenue for future developments in GBM [126]. Taken together, these preclinical findings warrant investigation in patients with GBM, which may improve the effectiveness of immunotherapy for this disease [127]. Despite some encouraging findings, the foremost limitation impeding CAR T therapy for GBM is the heterogeneity in GBM, which make it difficult to develop CAR-based strategies that can target all of the clonal populations [78]. While this approach requires further validation, modifications to mitigate tumor antigen escape and overcome antigenic heterogeneity might provide a means for effective application of CAR T therapy for GBM treatment.

### Oncolytic virotherapy

The concept of virotherapy for malignancies was first demonstrated in a case report in 1912, when DePace described a woman with cervical cancer showed tumor regression after receiving an attenuated rabies virus vaccine. Since then, case studies reporting cancer remission in patients treated with naturally occurring viruses, especially in leukemias and lymphomas [128–130]. However, concerns of serious adverse events and the advent of chemotherapy halted early progress of oncolytic virotherapy [131]. Its potential was re-evaluated until the end of the twentieth century, supported by the evolution of viral molecular biology, as well as the development of reverse genetics system allowing for virus engineering [132]. GBM is particularly suitable for oncolytic virus (OV) therapy due to the tumor’s confinement to the brain, lack of distant metastases, and growth being surrounded mainly by post-mitotic cells, which allows for the use of viruses that require active cells cycles for replication [133].

Now, oncolytic virotherapy represents a promising form of immunotherapy for GBM treatment, which can be divided into two groups: 1) replication-competent OVs that selectively infect and replicate in cancer cells to kill tumor cells; and 2) replication-deficient viral vectors used as delivery vehicles for therapeutic genes. Currently, specific OVs have been genetically engineered to target pathogen-associated receptors present on tumor cells in order to achieve efficient and selective replication. The viral infection and amplification eventually elicit host antitumor immune responses and eliminate cancer cells. To date, over 20 oncolytic virus candidates including Herpes simplex virus Type 1 (HSV-1) [134–136], Adenovirus (Ad) [137], Reovirus [138], measles virus (MV) [139, 140], Newcastle disease virus [141], and Poliovirus [142] have been tested in clinical trials to treat GBM (Table 1). In addition, new developments have been also made in delivery techniques for OVs to overcome limitations imposed by the BBB. Recently, Desjardins et al. reported a novel technique for convection enhanced delivery (CED) of the recombinant nonpathogenic polio-rhinovirus chimera (PVSRIPO) [143]. PVSRIPO is a live attenuated poliovirus type 1 (Sabin) vaccine with its cognate internal ribosome entry site replaced with that of human rhinovirus type 2 in order to restrict neurovirulence. PVSRIPO targeted GBM through CD155, a high-affinity ligand for the T-cell immunoreceptor with immunoglobulin and immunoreceptor tyrosine-based inhibition motif domains, which is broadly upregulated on malignant cells. In the phase I trial (NCT01491893), intratumoral CED of PVSRIPO in patients with recurrent GBM confirmed the absence of neurovirulent potential, and was granted breakthrough-therapy designation by FDA in May 2016. Preliminary data have revealed that the survival rate among patients who received PVSRIPO immunotherapy was higher at 24 and 36 months than the rate among historical controls. On the basis of phase I findings, the phase II randomized trial (NCT02986178) of PVSRIPO alone or in combination with single-cycle lomustine in patients with recurrent GBM are underway. The therapeutic efficacy of this novel treatment modality in patients with GBM is eagerly awaited.

**Table 1:** Summary of clinical trials of oncolytic viral therapy for patients with glioblastoma

Treatment	Status	Enrolled Patients	Primary outcome measures	NCT number
Genetically Engineered Adenovirus DNX-2440	Recruiting phase I	24	Safety, OS, and ORR	NCT03714334
Combination of modified vaccinia virus TG6002 and 5-FC	unknown phase I/II	78	DLTs and tumor progression at 6 months	NCT03294486
Adenovirus DNX-2401 ± IFN-γ	Completed phase I	37	ORR by interval tumor size	NCT02197169
DNX2401 and TMZ	Completed phase I	31	Number of patients with AEs	NCT01956734
Genetically Engineered HSV-1 MVR-C5252 (C5252)	Not yet recruiting phase I	51	Safety and tolerability DLTs and MTD	NCT05095441
New Castle Disease Virus	Study withdrawn for unknown reasons phase I/II	0	PFS	NCT01174537
Recombinant nonpathogenic polio-rhinovirus chimera (PVSRIPO) administered by CED into tumor	Active, not recruiting phase I	61	MTD, DLTs and RP2D	NCT01491893
Adenovirus DNX-2401 and surgery	Recruiting phase I	36	MTD and Incidence of AEs	NCT03896568
Genetically Engineered HSV-1 G207	Completed phase I/II	65	Not provided	NCT00028158
Replication-competent Adenovirus (Delta-24-RGD) administered by CED into tumor	Completed phase I/II	20	treatment related serious AEs	NCT01582516
Neural stem cells (NSCs) loaded with an oncolytic adenovirus	Active, not recruiting phase I	13	maximum number of NSCs loaded with oncolytic adenovirus	NCT03072134
H-1 Parvovirus (H-1PV)	Completed phase I/II	18	Safety and tolerability	NCT01301430
HSV-1 mutant HSV1716	Study terminated for unknown reasons phase I	2	MTD	NCT02031965
Combination Adenovirus change NCT02798406 DNX2401 and pembrolizumab	Completed phase II	49	ORR by interval tumor size	
AdV-tk (adenoviral vector expressing HSV-TK) plus valacyclovir (antiviral drug) and SOC	Completed phase II	52	Safety and OS	NCT00589875
Genetically Engineered HSV-1 C134	Recruiting phase I	24	Safety and tolerability	NCT03657576
Oncolytic viral vector rQNestin34.5v.2	Recruiting phase I	56	MTD	NCT03152318
PVSRIPO	Active, not recruiting phase I	12	Toxicity within 14 days after PVSRIPO treatment	NCT03043391
Genetically Engineered HSV-1 M032	Recruiting phase I	36	MTD	NCT02062827
Single dose of G207 infused through catheters into tumors	Not yet recruiting phase II	30	OS	NCT04482933
PVSRIPO administered by CED into tumor	Active, not recruiting phase II	122	ORR rate and DORR at 24 and 36 months	NCT02986178
Single dose of G207 infused through catheters into tumors	Recruiting phase I	15	Safety and tolerability	NCT03911388
Single dose of G207 infusedthrough catheters into tumors	Active, not recruiting phase I	12	Safety and tolerability	NCT02457845
Live, replication-competent wild-type reovirus REOLYSIN®	Completed phase I	18	MTD, DLTs and 6- month response rate	NCT00528684
Combination of PVSRIPO and atezolizumab	withdrawn Re-submission Planned phase I/II	0	AEs within 14 days after atezolizumab treatment, proportion patients alive at 24 months after PVSRIPO infusion	NCT03973879
Toca511 & Toca FC versus SOC	Study terminated for unknown reasons phase II/III	403	OS	NCT02414165

Most data were obtained from findings from www.clinicaltrials.gov using the search terms “glioblastoma” and “oncolytic”; *5-FC* 5-flucytosine, *TMZ* temozolomide, *OS* overall survival, *ORR* objective response rate, *IFN-γ* interferon Gamma, *SOC* Standard of Care, *DLT* dose limiting toxicities, *AE* adverse event, *MTD* maximum tolerated dose, *PFS* progression-free survival, *HSV* herpes simplex virus, *CED* convection-enhanced delivery, *NSC* neural stem cells, *RP2D* recommended phase 2 dose, *ORR* objective radiographic response, *DORR* duration of objective radiographic response

Since the first application of virus engineering to an oncolytic HSV in murine glioblastoma models [144], the pace of clinical activities has accelerated considerably [145], with numerous ongoing or completed trials using modified HSV constructs (Table 1). Trials including G207 (NCT00028158, NCT03911388 and NCT02457845), HSV-1716 (NCT02031965), M032 (NCT02062827), MVR-C252 (NCT05095441), C134 (NCT03657576) have been conducted or are ongoing in patients with GBM. G47Δ, a third-generation oncolytic HSV-1, has been tested in a Phase I–IIa trial (UMIN-CTR: UMIN000002661) for GBM patients in Japan and demonstrated the safety of G47Δ inoculated into the human brain. The subsequent investigator-initiated phase II clinical trial (UMIN-CTR: UMIN000015995) in patients with GBM has recently been completed with good results [146]. Base on the positive results from this phase II trial, G47∆ (Delytact/Teserpaturev) got conditional and time-limited approval for the treatment of malignant gliomas in Japan at June 2021. Additionally, several phase I and II trials, including (NCT02197169), (NCT01956734), (NCT03896568), (NCT01582516), and (NCT02798406) using genetically engineered oncolytic adenovirus combined with standard-of-care or immune checkpoint blockade are currently ongoing for patients with GBM (Table 1) and are given expectations to bring positive outcomes. Adenoviruses have also been modified to aglatimagene besadenovec (AdV-tk), an adenoviral vector containing the HSV thymidine kinase gene, followed by an antiherpetic prodrug such as valacyclovir, which functions as toxic nucleotide analogue that can kill tumor cells [147]. This approach, termed gene-mediated cytotoxic immunotherapy, was reported to be safe in newly diagnosed malignant gliomas in the phase I b clinical trial [148]. Subsequently, the phase II trial (NCT00589875) have been conducted and demonstrated notably improved survival outcomes for malignant gliomas associated with AdV-tk-based therapy [147] (Table 1). The clinical trials proved the safety and efficacy of OV therapy for GBM, but very few progressed to phase III trials. Previously, a phase III trial ASPECT (registered with EudraCT, number 2004–000464-28) assessed the efficacy and safety of adenovirus-mediated gene therapy with sitimagene ceradenovec followed by intravenous ganciclovir in patients with newly diagnosed resectable GBM. The ASPECT found no significant effect on OS [149]. Recently, a phase III trial (NCT02414165) of Toca511 & Toca FC was terminated for unknown reasons. Toca 511 consists of a purified retroviral replicating vector encoding a modified yeast cytosine deaminase (CD) gene. The CD gene converts the 5-flucytosine (5-FC) to the anticancer drug 5-FU in tumor cells that have been infected by the Toca 511 vector. Notably, several phase III trials for cancer immunotherapies combined with OVs have shown clinical promise for diverse cancers [150]. Oncolytic virotherapy for GBM remains promising and may impact the future of patient care. Recent studies have shown that Zika virus (ZIKV) has oncolytic activity against GSCs, suggesting that engineering of ZIKV may provide a therapeutic modality against Glioblastoma [151–155]. As ZIKV selectively infects and kills GSCs relative to normal neuronal cells, it may be an option to serves as a candidate for GBM therapy. Of note, despite the general safety of OV application confirmed by preclinical and clinical trials, the moderate clinical efficacy has not yet matched the preclinical promise from laboratory experiments.

### Vaccine therapy

Cancer vaccine therapy has shown great promise with both preventive and therapeutic potentials [156, 157]. For GBM, cancer vaccines is designed to target tumor-associated antigens to induce an immune response against tumors. Given that GBM-specific antigens are rare, GBM antigen targets are most often tumor-associated antigens, which limiting patient inclusion. To date, only a few vaccination approaches have reached phase III clinical testing in patients with GBM, and numerous others are at earlier stages of clinical development. The best studied tumor-specific antigen is EGFRvIII, which is a constitutively active mutant form of EGFR only expressed in 25–30% of GBM [158].

Rindopepimut (also known as CDX-110), a peptide vaccine targeting EGFRvIII has been tested in several clinical trials. In three uncontrolled phase II studies, rindopepimut vaccination in GBM patients with gross total resection and chemoradiotherapy have provided evidence of improved median survival of 24 months compared with historical controls [159–161]. Following these encouraging findings, an international phase III trial (NCT01480479), ACT IV was conducted to further assess the efficacy of rindopepimut in newly diagnosed patients with EGFRvIII-positive GBM. Despite the strong anti-EGFRvIII immune response generated in patients, the primary study analysis did not show a survival benefit for patients with minimal residual disease who received rindopepimut with TMZ versus those who received TMZ alone [162]. Of note, the spontaneous loss of antigen was seen in both the treatment and control arm, questioning the utility of immunotherapy targeting a single tumor antigen with heterogeneous tumor expression [162]. Recent evidence from a double-blind, randomized, phase II study (NCT01498328) in a smaller cohort of patients with recurrent EGFRvIII-positive GBM suggested favorable outcomes for rindopepimut when combined with standard bevacizumab versus bevacizumab alone [163]. Taken together, the positive results with rindopepimut in recurrent GBM in ReACT and the negative results of ACT IV in newly diagnosed GBM lend support to further clinical trials that use combination strategies such as immunotherapy with angiogenesis inhibition.

ICT­107 is a six synthetic peptide stimulated DC vaccine specifically designed for GBM, which has also reached to phase III clinical trials. A phase I study demonstrated the safety of ICT-107 with a suggestion of benefit to patients who were HLA-A2 positive [164]. A phase II trial showed that ICT-107 has some therapeutic activity in HLA-A2 positive patients and led to a phase III trial (NCT02546102) in HLA-A2^+^ newly diagnosed patients with GBM. But this phase III trial was suspended in 2017 due to lack of funding. DCVax­L, a dendritic cell-based vaccine therapy which use whole tumor lysate to pulse patient-derived DCs. Given the promising result in preclinical models and early stage clinical trials [165, 166], a phase III trial (NCT00045968) of DCVax-L was conducted in newly diagnosed GBM. In this trial, the overall intent-to-treat population had a median OS of 23.1 months which is superior to median OS of 15–17 months from past studies and clinical practice [167]. However, this trial was subsequently dropped for unidentified reasons. To summarize, current results from the clinical trials on vaccines for GBM are not very promising, lack of GBM-specific antigen and high heterogeneity of the tumors pose challenges to GBM vaccine therapy.

Recently, advances in next-generation sequencing and novel bioinformatics tools have enabled the systematic discovery of tumor neoantigens, which are derived from somatic mutations of the tumor and are therefore tumor specific [157, 168]. Neoantigens are highly specific for individual patients and hence, tumor vaccines targeting neoantigens can effectively trigger de novo T cell responses against neoantigens, thereby achieving personalized precision treatment. Initial studies of personalized neoantigen-based vaccines have demonstrated robust tumor-specific immunogenicity and preliminary evidence of anti-tumor activity in patients with high-risk melanoma and other cancers [168]. Based on the encouraging findings, a phase I/Ib study of personalized neoantigen vaccines has been tested in 10 patients with newly diagnosed *MGMT*-unmethylated GBM following surgical resection and conventional radiotherapy. Patients who did not receive dexamethasone generated circulating polyfunctional neoantigen-specific CD4^+^ and CD8^+^ T cell responses that were enriched in a memory phenotype and showed an increase in the number of tumor-infiltrating T cells [169]. Despite generating systemic and intratumoral neoantigen-specific immune responses post-vaccination, all patients showed tumor recurrence and ultimately died of progressive disease, indicating that the induced T cell responses must still overcome considerable challenges to produce clinically relevant anti-tumor activity, including tumor-intrinsic defects and immunosuppressive factors in the microenvironment [169]. Given that neoantigen-targeting vaccines have the potential to favorably alter the immune milieu of glioblastoma, thus, combining vaccination with other regimens such as immune checkpoint inhibition may be beneficial.

### Focused ultrasound therapy

Despite incremental advances in the therapeutic approach to GBM, there has been minimal development of both new and existing drug therapies for recurrent GBM [6]. The last drug to significantly improve OS for GBM was TMZ, which was introduced 20 years ago [35]. After decades of development, bevacizumab, a humanized monoclonal antibody that inhibits vascular endothelial growth factor (VEGF) was granted accelerated FDA approval for recurrent GBM without the completion of a randomized Phase III trial, making bevacizumab the third FDA-approved treatment for GBM [170]. Subsequently, bevacizumab was tested in two large randomized phase III trials (NCT00884741 and NCT00943826) [10, 11]. Despite improvement in median progression-free survival (PFS) of both trials, first-line use of bevacizumab did not improve OS in patients with glioblastoma. Consistent with this, according to a systematic analysis, the combination of bevacizumab for newly diagnosed GBM is beneficial in terms of prolonging median PFS but not OS [171]. Thus, innovative therapies are needed to ultimately improve the outcome of patients with glioblastoma. One of the major limitations of new GBM therapies in part because of inefficient drug delivery across the BBB. The BBB is formed by brain endothelial cells lining the cerebral microvasculature, presents a particular challenge for drug delivery [34]. Recently, focused ultrasound to overcome the BBB has led to the emergence of this technology as a viable new option for targeted delivery to the CNS [172]. Preclinical studies have showed that low-intensity pulsed ultrasound increased the concentrations of systemically administered drug therapies in the brain parenchyma in animal models and prolonged survival in GBM preclinical models [31, 33, 173–177].

After several decades of pre-clinical research, focused ultrasound has recently translated into clinical studies for GBM [178]. In 2016, a first-in-man, single-arm, single-center trial (NCT02253212) was initiated to evaluate the safety and feasibility of repeated pulsed ultrasound in recurrent GBM [34]. The results showed that focused ultrasound as a new technique for treating patients with GBM was safe and not burdensome [34, 35]. More importantly, the pulsed ultrasound add-on treatment presented in this work can be extended and combined with other therapies to enhance drug penetration in patients with GBM [35]. A prospective single-arm, open-label trial was conducted to investigate serial magnetic resonance-guided focused ultrasound (MRgFUS) and adjuvant TMZ combination in patients with GBM (NCT03616860). This first-in-human proof-of-concept study showed that MRgFUS enriches the signal of circulating brain-derived biomarkers, providing data for the feasibility of a focused ultrasound framework to liquid biopsy in neuro-oncology patients [179]. Transient BBB opening in tumor using non-invasive low-intensity MRgFUS with systemically administered chemotherapy was reported to be safe and feasible (NCT02343991) [180].

In addition, several clinical trials including (NCT04998864), (NCT04988750), and (NCT04446416) to evaluate the safety and preliminary efficacy of focused ultrasound are underway.

## Conclusions and perspectives

Immunotherapy has already demonstrated safety and feasibility for a variety of malignancies, its efficacy in clinical trials for glioblastoma remain to be investigated. Currently, standard therapy consists of tumor resection followed by radiotherapy and concomitant TMZ are still the mainstay of treatment, all of which have immunosuppressive effects. Besides, the glioblastoma microenvironment is a hostile attribution for anti-tumor immune responses, we must be cognizant of this complexity when developing immunotherapies. Hence, combination approaches with the aim of making these “cold” tumors “hot” are urgently needed and thus augmenting current immunotherapy strategies. Although immunotherapy represents a rapidly developing frontier in GBM therapy, consistent and sustained responses remain rare. There are still many challenges including: (i) local immunosuppression in the microenvironment after treatments which made the efficacy being modest and limited to a minority of patients; (ii) deficiency of specific tumor antigens and high tumor heterogeneity within GBM; (iii) chronic immune toxicities and the long-term implications of these effects associated with immunotherapy. Despite the encouraging results of preclinical and phase I/II clinical trials, even successful in a few case reports, the phase II/III transition remains particularly challenging, no successful phase III clinical trials with large patient cohorts for GBM immunotherapy have been reported so far.

Given that immunotherapy and conventional treatment act on different targets, synergistic or combined treatment may achieve greater therapeutic outcomes. However, intense research and clinical development are required to optimize the available treatment options and to overcome potential side effects. The success of this strategy includes the use of validated biomarkers, appropriate patient selection criteria, strategies to prevent adverse events, and the implementation of immunotherapy in multimodal treatment approach together with conventional therapies. Immunotherapy strategies based on well-known checkpoint blockades have shown promising activity against GBM in preclinical models and some case reports, whereas the results emerging from clinical trials with large patient cohorts are disappointing. The main reason might be that multiple genomic and epigenetic features are involved in the development of GBM, which may determine the response pattern of patients with GBM to checkpoint blockade-based immunotherapy. Therefore, a deeper understanding of the molecular pathology of GBM, tumor-intrinsic dominant signaling pathways driving tumorigenesis that are candidates to become therapeutic targets and tumor-specific antigenic profiles more effectively are urgently needed. Given that the heterogeneity across patients often lead to failure with immunotherapy, adding other therapeutic modalities such as molecular targeted therapy to immunotherapy may create new avenues for success. Combining immune checkpoint therapy with these novel agents may even further clinical activity of the PD-1 and CTLA-4 blockades. In addition, targeting “next generation” checkpoints is warranted as a single agent or in combination with other immunomodulatory approaches for GBM. Future treatments will likely consist of checkpoint blockade with addition of individualized therapy on the basis of tumor subtype and site of metastatic disease.

CAR T-based immunotherapy represents a promising therapeutic approach, but antigenic heterogeneity and restoration of immunosuppressive milieu post-therapy may limit the durability of responses to CAR T therapy. Identification of stably expressed and sufficiently tumor-specific antigens and agents that target immunosuppressive molecules are required to overcome the barrier. Recently, BiTEs have been tested in preclinical studies as a solution against antigen escape, it remain to be determined to successfully translate the new molecular findings into improved clinical management. Oncolytic viruses might exert pro-inflammatory responses, thus providing a potential to overcome the immunosuppression of glioblastoma. The future direction of oncolytic viral therapies seems to be focused on combinations with other immunotherapy strategies, in the hope of exploiting the potentially durable anticancer immune responses initiated by the viral infection to elicit prolonged clinical responses. Based on this, a combination of CAR T and OVs may benefit mutually. OV infection induces local inflammation and attracts T cells to tumors, which can reinforce the attraction of CAR T cells in TME [181]. Despite the promise of this combination approach, the main impediment to this strategy is the rapid clearance of OVs, presenting a challenge to clinical practice in future [182]. Vaccine therapy has been considered one of the most promising approaches to improve the outcomes of patients with GBM, but data from the clinical trials GBM are disappointing. Given that the lack of high expression of GBM-specific antigens are limiting factors in the development of peptide vaccine-based strategies, personalized neoantigen-based vaccines have attracted much attention in GBM vaccine therapy, although its clinical efficacy requires further investigation. To summarize, the experiences that have been gathered with immunotherapy for GBM is generally insufficient to translate into significant clinical benefit, combinatorial approaches might provide superior results. Despite the challenges and disappointing clinical results existed in developing immunotherapy for GBM, pursuing this path is justified given not only the therapeutic potential of this treatment, but also given the accelerating rate of progress. Additionally, the clinical realities of the contribution of the BBB to treatment failure in GBM argue for renewed efforts to optimize BBB-disruption technologies, develop BBB-penetrating agents, and refine implantable drug delivery technologies that bypass the BBB [183].

## Data Availability

Not applicable.

## Abbreviations

GBM: Glioblastoma
WHO: World Health Organization
TMZ: Temozolomide
GSC: Glioma stem cell
BBB: Blood-brain barrier
CNS: Central nervous system
CAR-T: Chimeric antigen receptor T
MHC: Major Histocompatibility Complex
APC: Antigen presenting cells
DC: Dendritic cell
HLA: Human leukocyte antigen
MGMT: Methylguanine-DNA methyl-guanine-methyltransferase
CTLA-4: Cytotoxic-T-lymphocyte-associated protein 4
PD-1: Programmed cell death protein 1
PD-L1: PD-1 ligand 1
FDA: U.S. Food and Drug Administration
mAb: Monoclonal antibody
IDO: Indoleamine 2,3 dioxygenase 1
CD47: Cluster of differentiation 47
SIRPα: Signal regulatory protein α
AML: Acute myeloid leukemia
ATP: Adenosine triphosphate
eADO: Extracellular adenosine
ADPR: ADP-ribose
ScFv: Single-chain variable fragment
TM: Transmembrane
EGFRvIII: Epidermal growth factor receptor variant III
IL-13Ra2: Interleukin (IL)13Rα2
Her2: Ephrin-A2
VST: Virus-specific T
BiTEs: Bispecific T cell engagers
OV: Oncolytic virus
HSV-1: Simplex virus Type 1
Ad: Adenovirus
MV: Measles virus
CED: Convection enhanced delivery
CD: Cytosine deaminase
5-FC: 5-flucytosine
ZIKV: Zika virus
VEGF: Vascular endothelial growth factor
PFS: Progression-free survival
OS: Overall survival
MRgFUS: Magnetic resonance-guided focused ultrasound
